# Supplementary figures and images for: Genomic analysis of ST88 community-acquired methicillin resistant Staphylococcus aureus in Ghana
Source: PeerJ. 2017 Feb 28;5:e3047. doi: 10.7717/peerj.3047 (PMC5333547; doi:10.7717/peerj.3047)

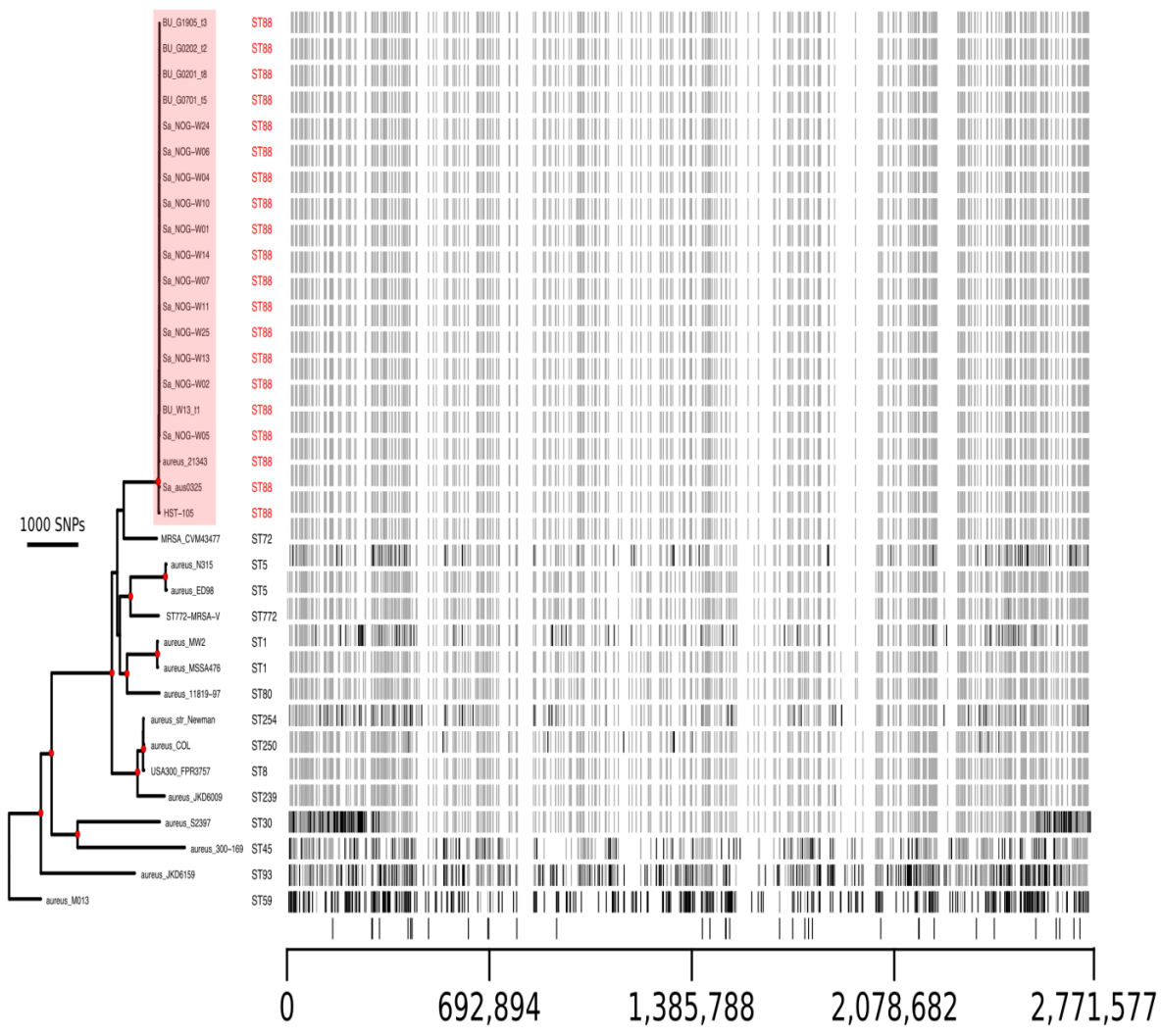

Supplement: Supplemental Information 1 — Light grey and black blocks denote recombination regions detected in ancestral nodes and the sampled genomes, respectively. In total there were 98,432 core SNPs, 26,570 of which were located within inferred regions of recombination. Red coloured isolates ST88, black; non-ST88 isolates. [file peerj-05-3047-s001.pdf]

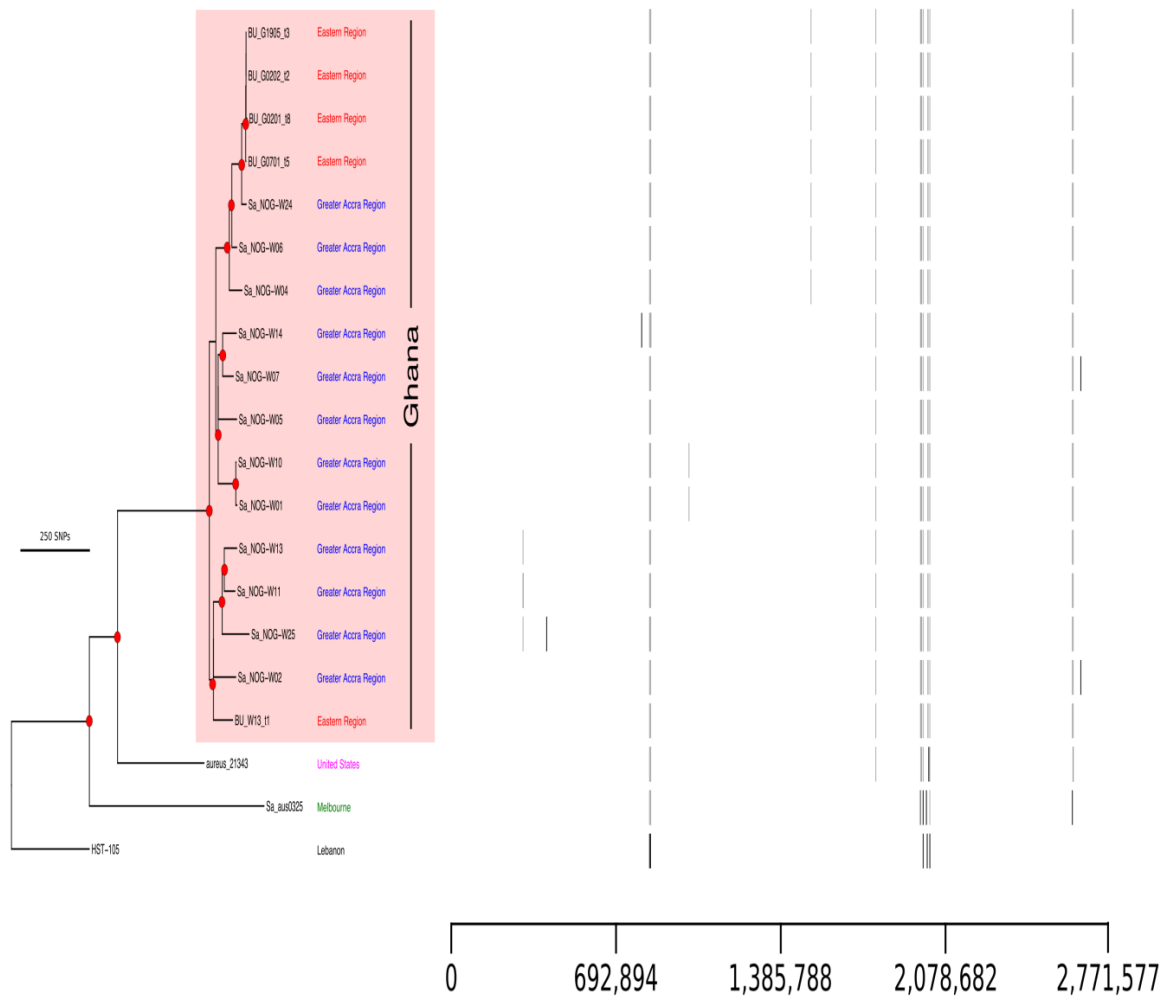

Supplement: Supplemental Information 2 — Light grey and black blocks denote recombination regions detected in ancestral nodes and the sampled genomes, respectively. In total there were 1,966 core SNPs, 207 of which were located within inferred regions of recombination. Blue and red coloured isolates from the Greater Accra and Eastern Region of Ghana respectively. Pink coloured isolate from the United States, Green from Melbourne and Black from Lebanon. [file peerj-05-3047-s002.pdf]
